# Association of the Insulinemic Potential of Diet and Lifestyle With Risk of Digestive System Cancers in Men and Women

**DOI:** 10.1093/jncics/pky080

**Published:** 2019-01-30

**Authors:** Weike Wang, Teresa T Fung, Molin Wang, Stephanie A Smith-Warner, Edward L Giovannucci, Fred K Tabung

## Abstract

**Background:**

We examined the role of the insulinemic potential of diet and lifestyle in the development of cancers of the digestive system, using two plasma C-peptide-based indices: the empirical dietary index for hyperinsulinemia (EDIH) and empirical lifestyle index for hyperinsulinemia (ELIH).

**Methods:**

We used Cox regression to analyze data on 45 816 men (Health Professionals Follow-up Study, 1986–2012) and 74 191 women (Nurses’ Health Study, 1984–2012) to examine associations between EDIH and ELIH scores and digestive system cancers. We computed the diet-only score (EDIH) from food-frequency questionnaires administered every 4 years. The lifestyle score (ELIH) included diet, body mass index, and physical activity. Outcomes included incident cancer of the digestive system (mouth, throat, esophagus, stomach, small intestine, and colorectum) and its accessory organs (pancreas, gallbladder, and liver). *P* values were two-sided.

**Results:**

We found direct associations between higher insulinemic potential of diet or lifestyle and risk of developing digestive system cancers in both men and women. The pooled multivariable hazard ratios (HRs) for participants comparing the highest to lowest EDIH quintile were: HR = 1.27, 95% confidence interval (CI) = 1.15 to 1.40, *P*_trend_ < .001 for digestive system cancers; HR = 1.30, 95% CI = 1.17 to 1.45, *P*_trend_ < .001 for digestive tract cancers (excluding accessory organs); and HR = 1.15, 95% CI = 0.93 to 1.41, *P*_trend_ = .48 for digestive accessory organ cancers. The same associations were stronger with the lifestyle score: HR = 1.47, 95% CI = 1.23 to 1.76, *P*_trend_ < .001 for digestive system cancers; HR = 1.49, 95% CI = 1.14 to 1.95, *P*_trend_ = .001 for digestive tract cancers; and HR = 1.43, 95% CI = 1.17 to 1.73, *P*_trend_ < .001 for digestive accessory organ cancers.

**Conclusions:**

The findings suggest that interventions to reduce the insulinemic potential of diet and lifestyle may be a means of preventing digestive system cancer.

Digestive system cancers include those of the digestive tract (mouth, throat, esophagus, stomach, small intestine, colorectum) and malignancies of digestive accessory organs (pancreas, gallbladder, liver). Digestive system cancers accounted for an estimated 18% of newly diagnosed cancers and 26% of cancer deaths in the United States in 2018 (1). Individual digestive system cancers are etiologically heterogeneous, yet evidence also suggests that common carcinogenic pathways may be operative. Specifically, proinflammatory pathways, inhibited by aspirin, appear to influence cancers specifically of the digestive tract (2). Physical inactivity and obesity also are associated with increased risk of digestive system cancers, among other cancers such as breast and endometrial cancer (3). Epidemiologic data strongly supports that persons with the metabolic syndrome and diabetes are at an increased risk of at least some digestive system cancers (4–8). The existence of specific factors for individual cancers (eg, *H. pylori* and gastric cancer) is indisputable. Nonetheless, that additional broadly acting factor(s) could influence risk of the entire digestive system is plausible.

The commonality of some of these factors, particularly obesity, physical inactivity, diabetes, and metabolic syndrome, is suggestive of a metabolic factor that may be strongly linked to these cancers (3). Evidence suggests that hyperinsulinemia may play a key role directly or by enhancing the bioavailability of insulin-like growth factor-1 (IGF-1), a stimulant for tumor growth and development (6,9–11). Moreover, plasma levels of insulin and C-peptide, markers of hyperinsulinemia, predict risk of digestive system cancers, including colorectal, gastric, and pancreatic cancers (1,2,6,12–20).

Given that hyperinsulinemia is considered an important risk factor for digestive system cancers, obesity and physical inactivity may increase risk of these cancers because they are major determinants of insulin resistance and hyperinsulinemia. Further, we would predict that dietary factors that influence hyperinsulinemia would also be associated with risk of these cancers. Thus, we sought to examine the association of the insulinemic potential of diet and lifestyle on risk of digestive system cancers. Compared with single nutrients or food items, a dietary pattern may more comprehensively affect insulin; we therefore previously developed an index to assess the insulinemic potential of whole diets, termed the empirical dietary index for hyperinsulinemia (EDIH) (21). We further computed an empirical lifestyle index for hyperinsulinemia (ELIH), which includes body mass index (BMI) and physical activity as components, in addition to diet. The current study focuses on the associations of EDIH and ELIH with digestive system cancers.

## Methods

### Study Populations

The Nurses’ Health Study (NHS) and Health Professionals Follow-up Study (HPFS) are ongoing prospective cohorts established in 1976 and 1986, respectively. The NHS (n = 121 701) enrolled female registered nurses ages 30–55 years, and the HPFS (n = 51 529) enrolled male health professionals ages 40–75 years. Since the inception of both cohorts, participants have completed self-administered questionnaires biennially, providing updated information on medical and lifestyle factors. Follow-up rates for both cohorts exceed 90% in each 2-year cycle.

For this analysis, we defined baseline as 1984 for NHS and 1986 for HPFS. At baseline, we excluded women and men with a history of any cancer except nonmelanoma skin cancer. Participants with excessive missing items (≥70) on the food-frequency questionnaires (FFQs) or implausibly low or high energy intake (<600 or >3500 kcal/d for women and <800 or >4200 kcal/d for men) were excluded. The final analysis included 74 191 women in the NHS and 45 816 men in the HPFS. The current study was approved by the institutional review boards at the Brigham and Women’s Hospital and at the Harvard T.H. Chan School of Public Health.

### Assessment of Covariate Data

Both cohorts collected nondietary data (eg, medical history and health practices) and updated the data through biennial self-administered questionnaires. We calculated participants’ BMI (kg/m^2^) using height (meters) reported at baseline for each cohort, and weight (kilograms) reported in each 2-year questionnaire cycle. Participants reported smoking status (never, former, current), and we calculated physical activity by summing the average metabolic equivalent-hours per week for each participant. Regular use of aspirin or other nonsteroidal anti-inflammatory drugs (NSAIDs) was defined as use of 2 or more standard tablets (325 mg) of aspirin or 2 or more tablets of NSAIDs per week. We derived a chronic disease comorbidity score by summing the presence equals 1 or absence equals 0, of the following chronic diseases or conditions: hypercholesterolemia, high blood pressure, heart disease, stroke, and rheumatoid or other arthritis.

### Calculation of the EDIH and the ELIH Scores

Dietary intake was assessed in 1984, 1986, and every 4 years thereafter in the NHS. In the HPFS, similar FFQs were initially administered in 1986 and subsequently every 4 years (20,22). The development of the EDIH and ELIH scores has been previously described (21). The goal was to create empirical scores to assess the potential of whole diets to stimulate insulin secretion. Briefly, 39 predefined food groups (22) were entered into stepwise linear regression analyses to identify the most important component food groups (EDIH) or in separate analyses, food groups and two lifestyle factors (BMI and physical activity) (ELIH), contributing to hyperinsulinemia. The 39 food groups were modeled as the independent variables predicting fasting plasma concentrations of C-peptide as a marker for insulin secretion, using a statistical significance level of *P *=* *.1 for entry into and retention in the stepwise linear regression model. The EDIH score is a weighted sum of 18 food groups; 13 were directly associated with C-peptide levels and five were inversely associated. The ELIH score is a weighted sum of 12 food groups, BMI, and physical activity. Six food groups and BMI were directly associated with C-peptide levels, and six food groups and physical activity inversely associated. Index components are presented in Supplementary Table 1. The weight for each index component was the corresponding beta coefficient from linear regression analyses. Higher (more positive) scores on both indices indicate hyperinsulinemic dietary patterns or lifestyles, and lower (more negative) scores indicate less insulinemic diets or lifestyles (21).

### Ascertainment of Incident Cancer

Our primary end point was incident digestive system cancer, which was reported through biennial follow-up questionnaires through 2012 for both cohorts. Study physicians unaware of participants’ exposure status reviewed medical records and confirmed self-reported cancer diagnosis. Digestive tract cancers included cancers of the mouth, throat, esophagus, stomach, small intestine, colon, and rectum. Digestive accessory organs included pancreas, gallbladder, and liver. Digestive system cancers were defined as cancers of the digestive tract and accessory organs.

### Statistical Analysis

Analyses were performed with the SAS statistical package version 9.4 for UNIX (SAS Institute, Cary, NC). Statistical tests were two-sided, and *P* < .05 was considered statistically significant.

We used Cox proportional hazards models to estimate hazard ratios (HR) and 95% confidence intervals (CI) for the associations of cancer incidence and index quintiles using the lowest quintile as the reference. Linear trends across index quintiles were evaluated using the median score of each quintile as an ordinal variable. Age (in months) was the underlying time scale. All models were stratified by age and calendar time. Participants contributed person-time from the return of the baseline questionnaire until diagnosis, death, or end of follow-up (January 31, 2012 for HPFS and June 1, 2012 for NHS), whichever occurred first. We examined proportionality of hazards for each covariate included in the Cox models using time by covariate interaction terms and found no violations (all *P* > .05). To address the concern of potential bias from reverse causation, that is, occult chronic diseases in the years that preceded diagnosis, may have influenced dietary intake; we used a 2-year lag between dietary intake assessment and gastrointestinal tract cancer diagnosis as the main analytic approach. For example, in the NHS, we used EDIH and ELIH scores from the 1984 questionnaire in relation to cancers diagnosed from 1986 to 1988, and scores from the 1986 questionnaire in relation to cancers diagnosed from 1988 to 1990, and so forth). To better represent long-term diet and to minimize within-person variation, we calculated cumulative averages of the scores and potential confounding variables.

In the primary multivariable-adjusted model, we further adjusted for race (white or nonwhite), family history of cancer (yes or no), history of endoscopy (yes or no), current multivitamin use (yes or no), physical activity (metabolic equivalent-h/wk, continuous), intakes of total calories (kcal/d, continuous), total alcohol intake (g/d, continuous), pack-years of smoking, regular aspirin use (yes or no), regular NSAID use (yes or no), and additionally in women for menopausal status and postmenopausal hormone use. Because BMI and diabetes mellitus may be potential intermediates in the EDIH analyses, our primary multivariable-adjusted analysis did not control for these variables. However, in secondary analyses, we added BMI (kg/m^2^, continuous) into the multivariable model. In additional analyses, we excluded diabetics from the models and stratified EDIH models by BMI (<25, ≥25 kg/m^2^) while adjusting for continuous BMI in the strata. *P* values for heterogeneity were calculated with the use of the Q statistic, and in the absence of heterogeneity, we pooled the hazard ratios from multivariable models in each cohort using a random-effects meta-analysis. Furthermore, we created forest plots of the multivariable-adjusted hazard ratios for total digestive system cancer risk as well as specific digestive system cancer sites for comparisons of the highest decile of the index score to the lowest decile.

## Results

In HPFS, 45 816 men contributed 975 810 person-years from 1988 to 2012 with 2170 incident cases of digestive system cancers (1716 digestive tract cancers and 454 digestive accessory organ cancers). In NHS, 74 191 women contributed 1 606 889 person-years from 1986 to 2012 with 2445 incident cases of digestive system cancers (1859 digestive tract cancers and 586 digestive accessory organ cancers). Therefore, our study included 120 007 men and women among whom 4615 digestive system cancers were diagnosed in 2 582 699 person-years of follow-up. Men and women with higher EDIH and ELIH scores tended to be younger, had a higher BMI, exercised less, and were diabetic (we note that BMI and physical activity are components of ELIH). They also were more likely to be current smokers, took fewer multivitamins, and were less likely to undergo endoscopy (Table 1). In general, the differences among participants comparing the highest quintile of ELIH to the lowest quintile were greater than those for the diet-only (EDIH) score in both men and women (Table 1).

**Table 1.** Distribution of participant characteristics (weighted by person-years) across the entire follow-up period in quintiles of the EDIH and the ELIH scores in the NHS (1984–2012) and the HPFS (1986–2012*,†,‡

Characteristic	NHS (women)			HPFS (men)		
	Quintile 1	Quintile 3	Quintile 5	Quintile 1	Quintile 3	Quintile 5
Median EDIH score	−1.32	−0.03	1.39	−1.34	−0.01	1.39
Age, y	65.2 (9.7)§	63.9 (9.6)	60.0 (9.2)	64.5 (11.0)	64.2 (11.0)	60.2 (10.6)
Alcohol drinkers, %	73.1	56.6	47.9	80.7	71.6	62.1
Total alcohol, drinks/wk¶, among drinkers	7.3 (7.6)	4.2 (5.4)	3.9 (5.9)	10.7 (10.0)	7.2 (7.7)	6.3 (7.6)
Current smoker, %	11.8	11.7	15.5	3.9	4.4	6.4
Regular aspirin use, yes, %	61.6	60.5	59.8	46.1	45.9	40.1
Family history of colorectal cancer, yes, %	26.6	25.5	23.7	17.5	18.2	15.5
History of endoscopy, yes, %	23.6	21.8	16.4	25.2	25.3	20.4
Multivitamin use, yes, %	60.1	55.6	45.3	53.3	51.1	43.9
Diabetes, yes, %	2.5	5.3	8.7	2.3	3.3	5.4
Total energy intake, kcal/d	1850 (4.1)	1667 (4.5)	1821 (4.6)	2106 (5.3)	1878 (5.1)	2087 (5.0)
Dietary fiber, g/d	21.0 (6.5)	19.2 (5.5)	16.7 (4.9)	26.3 (8.7)	23.3 (7.0)	19.7 (6.0)
Dietary calcium, mg/d	821 (3.2)	793 (3.2)	697 (2.9)	872 (3.9)	864 (3.9)	786 (3.5)
Vitamin D, IU/d	211 (1.8)	212 (1.8)	188 (1.7)	267 (1.0)	261 (1.0)	241 (136)
Whole grains, g/d	27.8 (19.1)	25.2 (18.4)	17.4 (15.2)	35.6 (24.7)	31.7 (21.8)	23.3 (18.1)
Physical activity, MET-h/wk	22.5 (25.4)	17.0 (20.2)	14.2 (17.7)	35.5 (29.7)	31.6 (26.7)	29.2 (25.6)
BMI, kg/m^2^	24.7 (4.0)	26.3 (4.6)	27.9 (5.8)	24.2 (5.9)	24.8 (6.3)	25.7 (7.0)
Overweight or obese, ≥25 kg/m^2^, %	43.9	58.7	69.4	41.2	47.9	56.9
Postmenopausal, %	89.2	86.8	74.3	NA	NA	NA
Hormone therapy use ever‖, %	68.8	68.0	62.7	NA	NA	NA
Median ELIH score	−1.26	−0.08	1.45	−1.25	−0.08	1.44
Age, y	62.0 (10.0)§	63.3 (9.9)	63.4 (9.5)	62.5 (11.4)	63.3 (11.0)	63.5 (10.5)
Alcohol drinkers, %	69.1	60.0	44.2	76.7	74.0	70.4
Total alcohol, drinks/wk¶, among drinkers	6.0 (6.7)	4.6 (5.8)	4.4 (6.8)	8.4 (8.5)	7.2 (7.6)	9.1 (10.0)
Current smoker, %	15.8	12.5	9.4	5.0	4.6	6.0
Regular aspirin use, yes, %	60.3	60.5	60.5	44.7	48.3	48.4
Family history of colorectal cancer, yes, %	25.9	25.3	24.8	18.9	18.9	18.7
History of endoscopy, yes, %	20.3	21.2	20.2	24.7	26.3	25.0
Multivitamin use, yes, %	55.9	54.6	51.3	55.4	53.7	49.6
Diabetes, yes, %	1.2	3.2	14.5	2.6	3.1	7.0
Total energy intake, kcal/d	1784 (4.4)	1721 (4.4)	1767 (4.0)	2032 (5.0)	1939 (5.6)	2022 (5.1)
Dietary fiber, g/d	20.1 (6.5)	19.0 (5.6)	18.3 (5.5)	25.7 (8.6)	23.3 (7.2)	21.2 (6.6)
Dietary calcium, mg/d	778 (2.4)	784 (3.3)	773 (2.7)	863 (3.0)	855 (3.6)	841 (3.1)
Vitamin D, IU/d	203 (1.7)	209 (1.6)	205 (1.4)	267 (1.5)	259 (1.5)	250 (141)
Whole grains, g/d	25.2 (19.0)	24.3 (18.2)	22.4 (17.6)	35.3 (24.6)	31.9 (21.6)	26.9 (19.5)
Physical activity, MET-h/wk	23.8 (27.4)	17.1 (19.6)	12.7 (15.9)	37.4 (32.5)	32.0 (28.0)	26.6 (25.4)
BMI, kg/m^2^	22.1 (2.6)	25.5 (2.6)	32.4 (5.4)	22.7 (3.5)	25.0 (4.1)	29.3 (6.1)
Overweight or obese, ≥25 kg/m^2^, %	16.9	58.6	97.0	15.2	50.0	78.4
Postmenopausal, %	81.6	84.9	85.8	NA	NA	NA
Hormone therapy use ever‖, %	70.9	68.5	59.4	NA	NA	NA

* Weighted by follow-up time (person-years) accrued by each participant. BMI = body mass index; EDIH = empirical dietary index for hyperinsulinemia; ELIH = empirical lifestyle index for hyperinsulinemia; HPFS = Health Professionals Follow-up Study; MET = metabolic equivalent; NA = not applicable; NHS = Nurses’ Health Study.

† EDIH and ELIH scores were adjusted for total energy intake using the residual method. In EDIH and, ELIH quintiles, lower scores indicate insulin-sensitive diets and lifestyles respectively, and higher scores indicate hyperinsulinemic diets and lifestyles, respectively.

‡ EDIH consists of the following food groups: red meat, low-energy beverages, cream soups, processed meat, margarine, poultry, butter, French fries, other fish, high-energy beverages, tomatoes, low-fat dairy, eggs, wine, and coffee. ELIH consists of the following food groups: margarine, liquor, cream soups, butter, red meat, fruit juice, coffee, whole fruit, wine, high-fat diary, snacks, salad dressing, and then additionally BMI and physical activity.

§ Mean (SD) (all such values).

‖ Among postmenopausal women.

¶ Total drinks per day was calculated from the sum of drinks per day of red wine, white wine, beer, and liquor. One drink was defined as 12 ounces of beer, 5 ounces of wine, or 1.5 ounces of spirits.

The EDIH score was directly associated with total digestive system cancer risk, which was largely driven by digestive tract cancers (Table 2). Among men, the multivariable HR comparing the highest quintile of EDIH with the lowest quintile was 1.27 (95% CI = 1.11 to 1.46, *P*_trend_ = .001) for total digestive system cancer and 1.33 (95% CI = 1.14 to 1.56, *P*_trend_ < .001) for digestive tract cancers. The corresponding result for digestive accessory organ cancers combined was HR = 1.08 (95% CI = 0.79 to 1.46, *P*_trend_ = .78). A similar trend of associations was observed among women, with multivariable-adjusted HR comparing the highest quintile of EDIH with the lowest quintile showing a 26% higher risk (HR = 1.26, 95% CI = 1.11 to 1.45, *P*_trend_ < .001) for digestive system cancers, a 28% higher risk (HR = 1.28, 95% CI = 1.10 to 1.49, *P*_trend_ = .001) for digestive tract cancers, and no association with risk for digestive accessory organ cancers (HR = 1.21, 95% CI = 0.91 to 1.61, *P*_trend_ = .17) (Table 2).

**Table 2.** Multivariable-adjusted HRs and 95% CIs for digestive system cancer risk in quintiles of the EDIH and scores among men and women*,†,‡

Anatomic location of cancer	Quintile 1 (reference)	Quintile 2	Quintile 3	Quintile 4	Quintile 5	*P*_trend_§
Total digestive system						
Men, cases (n = 2170)	438	433	468	416	415	
Men, HR (95% CI)	1.00	1.00 (0.87 to 1.14)	1.14 (0.99 to 1.30)	1.07 (0.93 to 1.23)	1.27 (1.11 to 1.46)	<.001
Women, cases (n = 2445)	487	513	505	478	452	
Women, HR (95% CI)	1.00	1.06 (0.93 to 1.20)	1.10 (0.97 to 1.24)	1.14 (1.00 to 1.30)	1.26 (1.11 to 1.45)	<.001
No. cases	927	949	973	897	868	
Pooled HR (95% CI)‖	1.00	1.03 (0.94 to 1.13)	1.11 (1.02 to 1.22)	1.11 (1.01 to 1.21)	1.27 (1.15 to 1.40)	<.001
Digestive tract						
Men, cases (n = 1716)	340	333	358	351	334	
Men, HR (95% CI)	1.00	1.00 (0.85 to 1.16)	1.13 (0.97 to 1.31)	1.17 (1.00 to 1.36)	1.33 (1.14 to 1.56)	<.001
Women, cases (n = 1859)	376	380	381	363	359	
Women, HR (95% CI)	1.00	1.04 (0.90 to 1.20)	1.09 (0.95 to 1.27)	1.13 (0.98 to 1.32)	1.28 (1.10 to 1.49)	<.001
No. cases	718	713	740	716	694	
Pooled HR (95% CI)	1.00	1.01 (0.91 to 1.13)	1.11 (1.00 to 1.23)	1.15 (1.03 to 1.28)	1.30 (1.17 to 1.45)	<.001
Mouth/pharynx to small intestine						
** **Men, cases (n = 488)	94	102	92	102	98	
** **Men, HR (95% CI)	1.00	1.08 (0.82 to 1.44)	1.03 (0.77 to 1.38)	1.18 (0.88 to 1.57)	1.29 (0.96 to 1.73)	.08
** **Women, cases (n = 427)	80	90	87	86	84	
** **Women, HR (95% CI)	1.00	1.18 (0.87 to 1.60)	1.24 (0.91 to 1.69)	1.33 (0.97 to 1.83)	1.47 (1.07 to 2.03)	.01
** **No. cases	174	192	179	188	183	
** **Pooled HR (95% CI)	1.00	1.13 (0.92 to 1.39)	1.12 (0.91, 1.39)	1.25 (1.01, 1.54)	1.37 (1.10, 1.70)	.003
Stomach						
** **Men, cases (n = 126)	17	32	22	30	25	
** **Men, HR (95% CI)	1.00	1.97 (1.08 to 3.58)	1.41 (0.74 to 2.68)	1.88 (1.02 to 3.46)	1.99 (1.06 to 3.76)	.06
** **Women, cases (n = 118)	21	23	27	23	24	
** **Women, HR (95% CI)	1.00	1.05 (0.58 to 1.92)	1.32 (0.74 to 2.37)	1.26 (0.68 to 2.31)	1.50 (0.81 to 2.78)	.16
** **No. cases	38	55	49	53	49	
** **Pooled HR (95% CI)	1.00	1.44 (0.78 to 2.67)	1.36 (0.88 to 2.10)	1.54 (1.00 to 2.37)	1.72 (1.11 to 2.68)	.02
Colorectum						
** **Men, cases (n = 1232)	247	232	266	250	237	
** **Men, HR (95% CI)	1.00	0.96 (0.80 to 1.15)	1.16 (0.97 to 1.38)	1.16 (0.97 to 1.39)	1.35 (1.12 to 1.62)	<.001
** **Women, cases (n = 1439)	298	291	294	280	276	
** **Women, HR (95% CI)	1.00	0.99 (0.84 to 1.17)	1.05 (0.89 to 1.24)	1.08 (0.92 to 1.28)	1.22 (1.03 to 1.45)	.01
** **No. cases	547	523	561	532	513	
** **Pooled HR (95% CI)	1.00	0.98 (0.87 to 1.11)	1.10 (0.97 to 1.24)	1.12 (0.99 to 1.27)	1.28 (1.13 to 1.45)	<.001
Digestive accessory organs						
Men, cases (n = 454)	98	100	110	65	81	
Men, HR (95% CI)	1.00	1.00 (0.75 to 1.33)	1.17 (0.88 to 1.54)	0.72 (0.53 to 1.00)	1.08 (0.79 to 1.46)	.78
Women, cases (n = 586)	121	133	124	115	93	
Women, HR (95% CI)	1.00	1.11 (0.87 to 1.43)	1.10 (0.85 to 1.42)	1.16 (0.89 to 1.51)	1.21 (0.91 to 1.61)	.17
No. cases	219	236	233	181	164	
Pooled HR (95% CI)	1.00	1.06 (0.88 to 1.28)	1.13 (0.94 to 1.37)	0.92 (0.58 to 1.47)	1.15 (0.93 to 1.41)	.48
Pancreas						
** **Men, cases (n = 346)	79	78	81	48	60	
** **Men, HR (95% CI)	1.00	0.93 (0.68 to 1.28)	1.03 (0.75 to 1.41)	0.64 (0.44 to 0.92)	0.94 (0.66 to 1.33)	.27
** **Women, cases (n = 494)	103	119	94	99	79	
** **Women, HR (95% CI)	1.00	1.22 (0.93 to 1.59)	1.01 (0.76 to 1.35)	1.21 (0.91 to 1.61)	1.23 (0.90 to 1.67)	.23
** **No. cases	182	200	174	148	139	
** **Pooled HR (95% CI)	1.00	1.08 (0.83 to 1.40)	1.02 (0.82 to 1.26)	0.89 (0.47 to 1.67)	1.08 (0.83 to 1.42)	.97
Liver and gallbladder						
** **Men, cases (n = 108)	19	22	29	17	21	
** **Men, HR (95% CI)	1.00	1.28 (0.68 to 2.39)	1.79 (0.99 to 3.24)	1.11 (0.57 to 2.16)	1.70 (0.89 to 3.23)	.17
** **Women, cases (n = 169)	37	31	36	36	29	
** **Women, HR (95% CI)	1.00	0.84 (0.52 to 1.36)	1.03 (0.65 to 1.65)	1.15 (0.71 to 1.85)	1.16 (0.69 to 1.93)	.39
** **No. cases	56	54	65	54	50	
** **Pooled HR (95% CI)	1.00	0.99 (0.66 to 1.48)	1.32 (0.77 to 2.25)	1.13 (0.77 to 1.67)	1.34 (0.90 to 2.00)	.11

* EDIH scores were adjusted for total energy intake using the residual method. Lower scores indicate insulin-sensitive diets, and higher scores indicate hyperinsulinemic diets. CI = confidence interval; EDIH = empirical dietary index for hyperinsulinemia; HR = hazard ration; NSAIDs = nonsteroidal anti-inflammatory drugs.

† Heterogeneity for risk was tested using duplication method cause-specific Cox regression analyses.

‡ All analyses were adjusted for the following potential confounding variables: race, family history of cancer, history of endoscopy, multivitamin use, total alcohol intake, physical activity, pack-years of smoking, regular aspirin use, regular NSAIDs use, and additionally for menopausal status and postmenopausal hormone use in women.

§ The *P* value for linear trend across EDIH quintiles was the *P* value of the ordinal variable constructed by assigning quintile medians to all participants in the quintile. Models for linear trend were adjusted for all covariates listed in footnote previously.

‖ HRs were pooled using random effects meta-analysis (all such values).

Compared with EDIH, the ELIH score was more strongly associated with total digestive system cancer risk, and similarly, this association was largely driven by digestive tract cancers (Table 3). Among men, the multivariable HR comparing the highest quintile of ELIH to the lowest quintile showed a 62% higher risk (HR = 1.62, 95% CI = 1.41 to 1.85, *P*_trend_ < .001) for digestive system cancer, a 71% higher risk (HR = 1.71, 95% CI = 1.47 to 1.99, *P*_trend_ < .001) for digestive tract cancers, and 33% higher risk (HR = 1.33, 95% CI = 1.00 to 1.78, *P*_trend_ = .02) for digestive accessory organ cancers. Among women, corresponding results were a 35% higher risk (HR= 1.35, 95% CI = 1.19 to 1.53, *P*_trend_ = .001) for digestive system cancers, 30% higher risk (HR = 1.30, 95% CI = 1.13 to 1.50, *P*_trend_ < .001) for digestive tract cancers, and 51% higher risk (HR = 1.51, 95% CI = 1.23 to 1.86, *P*_trend_ = .001) for digestive accessory organ cancers. Considering the digestive tract cancers, ELIH was most strongly associated with colorectal cancer in men and women.

**Table 3.** Multivariable-adjusted HRs and 95% CIs for digestive system cancer risk in quintiles of the ELIH scores among men and women*^,^†^,^‡

Anatomic location of cancer	Quintile 1 (reference)	Quintile 2	Quintile 3	Quintile 4	Quintile 5	*P*_trend_§
Total digestive system						
Men, cases (n = 2170)	358	367	410	435	600	
Men, HR (95% CI)	1.00	1.03 (0.89 to 1.19)	1.12 (0.97 to 1.29)	1.19 (1.03 to 1.37)	1.62 (1.41 to 1.85)	<.001
Women, cases (n = 2445)	427	420	484	524	590	
Women, HR (95% CI)	1.00	0.98 (0.86 to 1.12)	1.08 (0.95 to 1.23)	1.14 (1.00 to 1.30)	1.35 (1.19 to 1.53)	<.001
No. cases	927	949	973	897	868	
Pooled HR (95% CI)‖	1.00	1.00 (0.91 to 1.11)	1.09 (0.99 to 1.21)	1.16 (1.06 to 1.28)	1.47 (1.23 to 1.76)	<.001
Digestive tract						
Men, cases (n = 1716)	275	287	326	345	483	
Men, HR (95% CI)	1.00	1.05 (0.89 to 1.24)	1.15 (0.98 to 1.36)	1.23 (1.05 to 1.45)	1.71 (1.47 to 1.99)	<.001
Women, cases (n = 1859)	337	308	379	387	448	
Women, HR (95% CI)	1.00	0.92 (0.79 to 1.07)	1.07 (0.93 to 1.24)	1.08 (0.93 to 1.25)	1.30 (1.13 to 1.50)	<.001
No. cases	718	713	740	716	694	
Pooled HR (95% CI)	1.00	0.98 (0.86 to 1.11)	1.11 (0.99 to 1.24)	1.15 (1.01 to 1.31)	1.49 (1.14 to 1.95)	.001
Mouth/pharynx to small intestine						
Men, cases (n = 488)	73	91	105	88	131	
Men, HR (95% CI)	1.00	1.21 (0.89 to 1.66)	1.41 (1.04 to 1.90)	1.18 (0.86 to 1.61)	1.64 (1.23 to 2.20)	.002
Women, cases (n = 427)	81	56	87	94	109	
Women, HR (95% CI)	1.00	0.72 (0.51 to 1.02)	1.07 (0.79 to 1.45)	1.13 (0.84 to 1.53)	1.39 (1.03 to 1.86)	.001
No. cases	174	192	179	188	183	
Pooled HR (95% CI)	1.00	0.94 (0.57 to 1.57)	1.23 (0.94 to 1.61)	1.15 (0.93 to 1.43)	1.51 (1.23 to 1.86)	<.001
Stomach						
Men, cases (n = 126)	19	27	28	21	31	
Men, HR (95% CI)	1.00	1.44 (0.80 to 2.61)	1.45 (0.80 to 2.61)	1.18 (0.63 to 2.22)	1.62 (0.90 to 2.89)	.21
Women, cases (n = 118)	21	23	27	23	24	
Women, HR (95% CI)	1.00	0.64 (0.32 to 1.30)	1.05 (0.57 to 1.92)	1.13 (0.62 to 2.05)	1.64 (0.94 to 2.87)	.01
No. cases	38	55	49	53	49	
Pooled HR (95% CI)	1.00	0.99 (0.45 to 2.17)	1.24 (0.81 to 1.88)	1.15 (0.75 to 1.78)	1.63 (1.09 to 2.44)	.007
Colorectum						
Men, cases (n = 1232)	203	198	220	258	353	
Men, HR (95% CI)	1.00	0.99 (0.82 to 1.21)	1.06 (0.87 to 1.28)	1.26 (1.04 to 1.52)	1.74 (1.46 to 2.07)	<.001
Women, cases (n = 1439)	257	253	293	294	342	
Women, HR (95% CI)	1.00	0.98 (0.82 to 1.17)	1.08 (0.91 to 1.27)	1.06 (0.90 to 1.26)	1.28 (1.09 to 1.51)	<.001
No. cases	547	523	561	532	513	
Pooled HR (95% CI)	1.00	0.99 (0.87 to 1.12)	1.07 (0.94 to 1.21)	1.15 (0.97 to 1.36)	1.49 (1.10 to 2.01)	.02
Digestive accessory organs						
Men, cases (n = 454)	83	80	84	90	117	
Men, HR (95% CI)	1.00	0.96 (0.70 to 1.30)	1.02 (0.75 to 1.38)	1.07 (0.79 to 1.45)	1.33 (1.00 to 1.78)	.02
Women, cases (n = 586)	90	112	105	137	142	
Women, HR (95% CI)	1.00	1.21 (0.92 to 1.60)	1.10 (0.83 to 1.46)	1.37 (1.04 to 1.79)	1.51 (1.15 to 1.98)	.001
No. cases	219	236	233	181	164	
Pooled HR (95% CI)	1.00	1.09 (0.86 to 1.37)	1.06 (0.86 to 1.30)	1.22 (0.97 to 1.55)	1.43 (1.17 to 1.73)	<.001
Pancreas						
Men, cases (n = 346)	66	66	66	66	82	
Men, HR (95% CI)	1.00	0.99 (0.70 to 1.40)	0.99 (0.70 to 1.39)	0.97 (0.69 to 1.38)	1.16 (0.84 to 1.62)	.37
Women, cases (n = 494)	77	94	94	99	130	
Women, HR (95% CI)	1.00	1.21 (0.90 to 1.64)	1.19 (0.88 to 1.61)	1.18 (0.88 to 1.60)	1.69 (1.27 to 2.26)	<.001
No. cases	182	200	174	148	139	
Pooled HR (95% CI)	1.00	1.11 (0.88 to 1.39)	1.10 (0.87 to 1.38)	1.09 (0.87 to 1.36)	1.42 (0.98 to 2.04)	.047
Liver and gallbladder						
Men, cases (n = 108)	17	14	18	24	35	
Men, HR (95% CI)	1.00	0.83 (0.40 to 1.69)	1.12 (0.57 to 2.18)	1.48 (0.79 to 2.78)	2.02 (1.12 to 3.65)	.002
Women, cases (n = 169)	26	28	24	51	40	
Women, HR (95% CI)	1.00	1.03 (0.60 to 1.77)	0.83 (0.48 to 1.46)	1.74 (1.08 to 2.80)	1.41 (0.85 to 2.33)	.03
No. cases	56	54	65	54	50	
Pooled HR (95% CI)	1.00	0.95 (0.62 to 1.46)	0.94 (0.61 to 1.44)	1.64 (1.12 to 2.40)	1.64 (1.11 to 2.40)	.001

* ELIH scores were adjusted for total energy intake using the residual method. Lower scores indicate insulin sensitive lifestyles, and higher scores indicate hyperinsulinemic lifestyles. CI = confidence interval; ELIH = empirical lifestyle index for hyperinsulinemia; HR = hazard ratio; NSAIDs = nonsteroidal anti-inflammatory drugs.

† Heterogeneity for risk was tested using duplication method cause-specific Cox regression analyses.

‡ All analyses were adjusted for the following potential confounding variables: race, family history of cancer, history of endoscopy, multivitamin use, total alcohol intake, pack-years of smoking, regular aspirin use, regular NSAIDs use, and additionally for menopausal status, and postmenopausal hormone use in women.

§ The *P* value for linear trend across ELIH quintiles was the *P* value of the ordinal variable constructed by assigning quintile medians to all participants in the quintile. Models for linear trend were adjusted for all covariates listed in footnote previously.

‖ HR were pooled using random effects meta-analysis (all such values).

**Figure 1.**
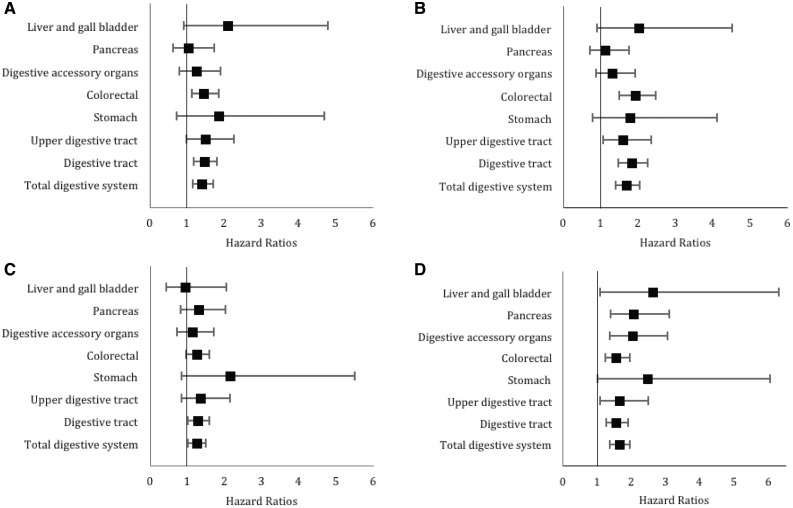
Forest plots of multivariable-adjusted hazard ratios and 95% confidence intervals for digestive system cancer risk comparing the highest decile to the lowest decile of (**A**) the empirical dietary index for hyperinsulinemia (EDIH) and (**B**) the empirical lifestyle index for hyperinsulinemia (ELIH) scores among men. **C**) EDIH and **D**) ELIH scores among women. All analyses were conducted using Cox regression and adjusted for the following potential confounding variables: race, family history of cancer, history of endoscopy, multivitamin use, total alcohol intake, pack-years of smoking, regular aspirin use, regular nonsteroidal anti-inflammatory drug use, and additionally for physical activity in EDIH models. In women, menopausal status and postmenopausal hormone use were additionally adjusted for.

For both EDIH and ELIH scores, the age-adjusted and multivariable results were similar. The results for the diet-only score (EDIH) did not change appreciably after additional adjustment for BMI (Supplementary Table 2) or after stratifying by BMI categories (Supplementary Tables 3 and 4). Also, results remained robust after excluding men and women who reported having diabetes (Supplementary Tables 5 and 6). To provide a stronger contrast, we compared the most adherent (highest decile) to the least adherent (lowest decile of EDIH and ELIH) to a hyperinsulinemic dietary pattern or lifestyle. The results for ELIH and EDIH were similar, but ELIH showed an overall higher risk for total digestive system (Figure 1).

There was no statistically significant heterogeneity by cohort (sex) across ELIH and EDIH quintiles for total digestive systems cancer or by cancer subsite (*P* for heterogeneity by sex for total digestive system cancer was 0.94 [EDIH] and 0.66 [ELIH]); therefore, we pooled the results from these independent cohort studies. The pooled multivariable HRs for participants, comparing the highest quintile of EDIH to the lowest, were: HR = 1.27, 95% CI = 1.15 to 1.40, *P*_trend_ < .001 for digestive system cancers; HR = 1.30, 95% CI = 1.17 to 1.45; *P*_trend_ < .001 for digestive tract cancers; and 1.15, 95% CI = 0.93 to 1.41, *P*_trend_  = .48 for digestive accessory organ cancers. Of the digestive tract cancers, the association between EDIH and colorectal cancer was the strongest (HR comparing highest to lowest quintile of 1.28; 95% CI = 1.13 to 1.45; *P*_trend_ < .001 and statistically significant associations were observed for upper digestive tract and stomach cancers (Table 2). The pooled multivariate HRs for participants comparing the highest quintile of ELIH to the lowest were HR = 1.47 (95% CI = 1.23 to 1.76, *P*_trend_ < .001) for digestive system cancers, HR = 1.49 (95% CI = 1.14 to 1.95, *P*_trend_ = .001) for digestive tract cancers, and HR = 1.43 (95% CI = 1.17 to 1.73, *P*_trend_ < .001) for digestive accessory organ cancers. Except for stomach and pancreatic cancer, ELIH was strongly associated with all other digestive system cancers (Table 3).

## Discussion

In two large prospective US cohorts, we evaluated the association of dietary (EDIH) and lifestyle (ELIH) indices developed to assess the insulin secretion potential of dietary and lifestyle behaviors and risk of developing cancers of the digestive system and its accessory organs. Our findings showed that higher scores on both indices, indicating a higher insulinemic potential of diet and lifestyle, were associated with higher risk of total digestive system cancers and digestive tract cancers, especially colorectal cancer. In general, associations for the lifestyle score were stronger than for the diet-only score, which fits with the explanation that in general, lifestyle is a stronger predictor of insulin response than diet alone. There was a 27% higher risk of developing digestive system cancer among men and women (combined) who were consuming the most hyperinsulinemic diets and a 47% higher risk of developing digestive system cancer among men and women with the most hyperinsulinemic lifestyles (diet, body weight, and physical inactivity).

Our overall results are consistent with previous findings of high insulin or C-peptide concentration on elevated risk for colorectal or colon cancer (23–28). Our results from NHS are also consistent with two previous studies in the same cohorts. Fung et al. (29) and Tabung et al. (30) found a direct associations between a dietary pattern predictive of C-peptide levels and colorectal cancer risk. The current study expanded on these previous findings and examined associations between diet and lifestyle scores and risk of developing digestive system cancers. In several nested case control studies, nonfasting C-peptide and low circulating IGF binding protein-1 (inhibits IGF-1 activity) were directly related to pancreatic cancer risk (31–34). Similarly, in our analysis, we found statistically significant associations between ELIH and pancreatic cancer risk. A recent nested case-control study in Japan found that higher insulin and C-peptide levels elevated risk of gastric cancer in men but not in women (17). Interestingly, we found that for stomach cancer, EDIH was similarly associated in women and men and statistically significantly associated in the pooled analysis. The lifestyle score, however was more strongly associated with stomach cancer risk in women than in men.

Because BMI and diabetes can be considered mediators of the diet-cancer association, we did not control for either in the primary analysis for the EDIH score. In our secondary analysis, the direct association between EDIH and digestive system cancers in both cohorts proved strong and independent of BMI, suggesting that EDIH is capturing additional information about the association between diet and cancer risk. The main mechanistic pathways that involve obesity are systemic inflammation and the insulin-IGF-glucose axis, and the pathways that do not directly involve obesity may include oxidative stress, compromised DNA repair, altered gut microbiome, and diminished immune function (35).

Our findings are compatible with a model linking modifiable factors (adiposity, physical activity, dietary pattern) with insulin, or closely related metabolic factors, and risk of digestive system cancers. Although diet, physical activity, and adiposity can be viewed as independent corroborative lines of evidence, they likely are biologically interactive. Why diverse organs of the digestive system may be affected by a common factor is not obvious at this point. However, common factors related to energy balance, such as insulin, may regulate overall proliferative activity of the digestive system in response to dietary intake and energy balance in a coordinated way. Chronically excessive stimulation of this pathway through energy imbalance may predispose to cancer risk (6,8,11,36,37).

Our study is not without limitations. Because the EDIH and ELIH indices were empirically derived from C-peptide data, the strength of the association between the scores and digestive system cancers depends not only on the association of index and biomarkers but also on the strength of association between C-peptide and digestive system cancers. However, in the validation studies, the EDIH and ELIH scores statistically significantly predicted biomarker concentrations (21,38). Other limitations of our study include its observational nature, our inability to entirely exclude all confounding factors (though most major risk factors for digestive cancer were included), and the self-reporting of dietary and lifestyle information may result in measurement error. However, to reduce random measurement error, the EDIH and ELIH indices were cumulatively averages from multiple time points, which is likely more relevant to the natural course of cancer that spans several decades. Moreover, although residual confounding is possible, the large effect sizes of our multivariable associations are unlikely to be entirely due to confounding. Furthermore, validation studies have shown reasonably good correlations between FFQ and diet reports, suggesting that dietary intake is well measured (22,39,40). Major strengths of this study include its prospective design, large samples, long follow-up, and serial updating of diet and lifestyle variables.

In conclusion, our analysis within two large prospective cohorts of men and women showed that higher scores of two indices assessing the insulin secretion potential of dietary and lifestyle behaviors are associated with higher risk of developing digestive system cancers. Our findings warrant testing reductions in the insulinemic potential of diet and lifestyle as a means for preventing the development of digestive system cancers.

## Funding

Dr Fred K. Tabung was supported by National Cancer Institute grant numbers K99CA207736 and R00CA207736. The HPFS and NHS cohorts are supported by NIH grants UM1CA167552 (HPFS), P01 CA55075 (HPFS), UM1CA186107 (NHS), and P01 CA87969 (NHS). No funding agency was involved in the design and conduct of the study; collection, management, analysis, and interpretation of the data; preparation, review, or approval of the manuscript; and decision to submit the manuscript for publication.

## Notes

Affiliations of Authors: Department of Nutrition (WW, TTF, SASW, ELG, FKT) and Department of Epidemiology (WW, MW, SASW), Harvard T.H. Chan School of Public Health, Boston, MA; Department of Nutrition, Simmons University, Boston, MA (TTF); Channing Division of Network Medicine, Department of Medicine, Brigham and Women’s Hospital and Harvard Medical School, Boston, MA (MW, ELG); Division of Medical Oncology, Department of Internal Medicine, The Ohio State University College of Medicine, Columbus, OH (FKT); The Ohio State University Comprehensive Cancer Center – Arthur G. James Cancer Hospital and Richard J. Solove Research Institute, Columbus, OH (FKT).

All authors declare no conflict of interest.

We thank the participants and staff of the HPFS and NHS cohorts for their valuable contributions as well as the following state cancer registries for their help: AL, AZ, AR, CA, CO, CT, DE, FL, GA, ID, IL, IN, IA, KY, LA, ME, MD, MA, MI, NE, NH, NJ, NY, NC, ND, OH, OK, OR, PA, RI, SC, TN, TX, VA, WA, and WY. The authors assume full responsibility for analyses and interpretation of these data.

Drs Weike Wang, Edward L. Giovannucci, and Fred K. Tabung had full access to all the data in the study and take responsibility for the integrity of the data and the accuracy of the data analysis. Author contributions: WW, ELG, and FKT designed research; WW conducted research and performed statistical analysis; WW, TTF, MW, SAS, ELG, and FKT analyzed and interpreted the data and provided critical input; WW wrote the paper; FKT provided study oversight; all authors read and approved final content.

## Supplementary Material

Supplementary Data
