# Supplementary material for: Association of the Insulinemic Potential of Diet and Lifestyle With Risk of Digestive System Cancers in Men and Women
Source: JNCI Cancer Spectr. 2019 Jan 30;2(4):pky080. doi: 10.1093/jncics/pky080 (PMC6352613; doi:10.1093/jncics/pky080)
Supplement: Supplementary Data [file pky080_supp.pdf]

Supplementary Table 1. Components food groups (servings/day) of the indices to assess the insulinemic potential of diet and lifestyle; Nurses' Health Study; 1990

**Empirical dietary index for hyperinsulinemia (EDIH)**		**Empirical lifestyle index for hyperinsulinemia (ELIH)**	
**Food group**	**Weight**	**Food group**	**Weight**
Direct associations		*Direct associations*	
Red meat	.089	Body mass index (kg/m^2)	.433
Processed meat	.058	Margarine	.039
Low energy beverages	.057	Liquor	.035
Cream soups	.052	Cream soups	.035
Margarine	.044	Butter	.028
Poultry	.041	Fruit juice	.026
High energy beverages	.041	Red meat	.026
Butter	.039	*Inverse associations*	
French fries	.036	Whole fruit	.046
Other fish	.037	Coffee	.047
Low fat dairy	.028	Wine	.036
Eggs	.026	Physical activity (MET-hrs/wk)	.032
Tomatoes	.032	High fat diary	.032
Cruciferous vegetables	.024	Snacks	.026
Inverse associations		Salad dressing	.020
Wine	.093		
Coffee	.071		
High fat dairy	.026		
Green leafy vegetables	.026		
Whole fruits	.024		
Dark yellow vegetables	.024		
Snacks	.022		

Weights are beta coefficients derived from the final step of the stepwise linear regression models from which the index components were obtained. Each weight represents the contribution of the corresponding index component to the total weighted index; The food groups retained were defined as follows: **red meats** (beef, pork, lamb, hamburger); **processed meats** (processed meats, bacon, hot dogs); **low energy beverages** (low-energy cola, other low-energy carbonated beverages); **cream soup**s (chowder or cream soup); **margarine**; **poultry** (chicken or turkey with or without skin); **high energy beverages** (cola with sugar, other carbonated beverages with sugar, fruit punch drinks); **butter**; F**rench fries**; **other fish** (canned tuna, shrimp, lobster, scallops, fish and other seafood other than dark meat fish); **low-fat dairy products** (skim or low-fat milk, sherbet or ice milk, yogurt); **tomatoes** (fresh tomatoes, tomato juice, tomato sauce); **eggs**; **tomatoes** (tomatoes, tomato juice, tomato sauce); **cruciferous vegetables** (broccoli; coleslaw and uncooked cabbage; cooked cabbage; cauliflower; Brussels sprouts; kale, mustard, and chard greens; sauerkraut); **wine** (red wine, white wine); **coffee**; **high-fat dairy products** (whole milk, cream, sour cream, ice cream, cream cheese, other cheese); **green leafy vegetables** (spinach, iceberg or head lettuce, romaine or leaf lettuce); **whole fruit** (raisins or grapes, avocado, bananas, cantaloupe, watermelon, fresh apples or pears, oranges, grapefruit, strawberries, blueberries, peaches, apricots, plums); **dark yellow vegetables** (carrots, yellow (winter) squash, yams, sweet potatoes); **snacks** (potato chips or corn chips, crackers, popcorn); **liquor**; **butter**; f**ruit juice** (apple juice or cider, orange juice, grapefruit juice, other fruit juice); **salad dressing** (oil and vinegar salad dressing); **refined grains** (white bread, English muffins, bagels or rolls, muffins or biscuits, white rice, pasta, pancakes or waffles); **beer**; **coffee**; **tea**; **potatoes**; **other vegetables** (celery, mushrooms, green pepper, corn, mixed vegetables, eggplant, zucchini, alfalfa sprouts, cucumber); **whole grains** (cooked oatmeal, other cooked breakfast cereal, dark bread, brown rice, other grains, bran added to food, wheat germ).

Supplementary Table 2. Multivariable-adjusted hazard ratios and 95% confidence intervals for digestive system cancer risk in quintiles of the empirical dietary index for hyperinsulinemia (EDIH) scores among men and women with additional adjustment for body mass index[*,†, ‡]

	Quintile 1 (reference)	Quintile 2	Quintile 3	Quintile 4	Quintile 5	P-trend[§]
**Total Digestive System**						
Men, cases (n=2170)	438	433	468	416	415	
Men, HR (95% CI)	1.00	1.00 (0.88, 1.15)	1.15 (1.01, 1.31)	1.08 (0.94, 1.24)	1.30 (1.13, 1.49)	<.001
Women, cases (n=2445)	487	513	505	478	452	
Women, HR (95% CI)	1.00	1.05 (0.93, 1.19)	1.09 (0.96, 1.23)	1.12 (0.99, 1.28)	1.24 (1.08, 1.42)	<.001
**Digestive Tract**						
Men, cases (n=1716)	340	333	358	351	334	
Men, HR (95% CI)	1.00	1.00 (0.85, 1.16)	1.13 (0.97, 1.31)	1.17 (1.00, 1.36)	1.33 (1.14, 1.56)	<.001
Women, cases (n=1859)	376	380	381	363	359	
Women, HR (95% CI)	1.00	1.03 (0.89, 1.19)	1.09 (0.94, 1.26)	1.13 (0.97, 1.31)	1.26 (1.08, 1.47)	.002
**Mouth/pharynx to small intestine**						
Men, cases (n=488)	94	102	92	102	98	
Men, HR (95% CI)	1.00	1.10 (0.82, 1.46)	1.06 (0.79, 1.41)	1.21 (0.91, 1.61)	1.33 (0.99, 1.79)	0.04
Women, cases (n=427)	80	90	87	86	84	
Women, HR (95% CI)	1.00	1.19 (0.87, 1.62)	1.26 (0.92, 1.72)	1.36 (0.99, 1.87)	1.52 (1.10, 2.10)	.008
**Stomach**						
Men, cases (n=126)	17	32	22	30	25	
Men, HR (95% CI)	1.00	1.97 (1.08, 3.59)	1.46 (0.77, 2.79)	1.97 (1.07, 3.63)	2.10 (1.11, 3.97)	.04
Women, cases (n=118)	21	23	27	23	24	
Women, HR (95% CI)	1.00	1.04 (0.57, 1.90)	1.28 (0.71, 2.30)	1.20 (0.65, 2.22)	1.40 (0.75, 2.62)	.25
**Colorectum**						
Men, cases (n=1232)	247	232	266	250	237	
Men, HR (95% CI)	1.00	0.95 (0.80, 1.15)	1.15 (0.96, 1.37)	1.14 (0.95, 1.37)	1.32 (1.10, 1.59)	<.001
Women, cases (n=1439)	298	291	294	280	276	

Women, HR (95% CI)	1.00	0.99 (0.84, 1.17)	1.04 (0.88, 1.23)	1.07 (0.90, 1.26)	1.19 (1.00, 1.42)	.04
**Digestive Accessory Organs**						
Men, cases (n=454)	98	100	110	65	81	
Men, HR (95% CI)	1.00	1.02 (0.77, 1.35)	1.20 (0.91, 1.59)	0.75 (0.55, 1.04)	1.11 (0.82, 1.51)	.99
Women, cases (n=586)	121	133	124	115	93	
Women, HR (95% CI)	1.00	1.10 (0.86, 1.41)	1.08 (0.83, 1.39)	1.12 (0.86, 1.46)	1.15 (0.86, 1.54)	.34
**Pancreas**						
Men, cases (n=346)	79	78	81	48	60	
Men, HR (95% CI)	1.00	0.95 (0.69, 1.30)	1.06 (0.77, 1.46)	0.67 (0.46, 0.96)	0.97 (0.69, 1.38)	.41
Women, cases (n=494)	103	119	94	99	79	
Women, HR (95% CI)	1.00	1.20 (0.92, 1.57)	0.98 (0.73, 1.30)	1.15 (0.86, 1.53)	1.13 (0.83, 1.55)	.54
**Liver and Gallbladder**						
Men, cases (n=108)	19	22	29	17	21	
Men, HR (95% CI)	1.00	1.30 (0.69, 2.43)	1.81 (1.00, 3.28)	1.13 (0.58, 2.21)	1.75 (0.92, 3.33)	.14
Women, cases (n=169)	37	31	36	36	29	
Women, HR (95% CI)	1.00	0.83 (0.51, 1.34)	1.01 (0.63, 1.62)	1.11 (0.69, 1.80)	1.11 (0.66, 1.87)	.46

NSAIDs=non-steroidal anti-inflammatory drugs; EDIH=empirical dietary index for hyperinsulinemia.

[*]EDIH scores were adjusted for total energy intake using the residual method. Lower scores indicate insulin sensitive diets, and higher scores indicate hyperinsulinemic diets.

[†]Heterogeneity for risk was tested using duplication method cause-specific Cox regression analyses

[‡]All analyses were adjusted for the following potential confounding variables: race, family history of cancer, history of endoscopy, multivitamin use, total alcohol intake, physical activity, pack-years of smoking, regular aspirin use, regular NSAIDs use, BMI, and additionally for menopausal status, and postmenopausal hormone use in women.

[§]The p-value for linear trend across EDIH quintiles was the p-value of the ordinal variable constructed by assigning quintile medians to all participants in the quintile. Models for linear trend were adjusted for all covariates listed in footnote #3.

Supplementary Table 3. Multivariable-adjusted hazard ratios and 95% confidence intervals for digestive system cancer risk in quintiles of the empirical dietary index for hyperinsulinemia (EDIH) scores among normal weight (BMI < 25 kg/m^2) men and women[*,†,‡]

	Quintile 1 (reference)	Quintile 2	Quintile 3	Quintile 4	Quintile 5	P-trend[§]
**Total Digestive System**						
Men, cases (n=1164)	274	245	238	207	200	
Men, HR (95% CI)	1.00	0.97 (0.81, 1.15)	1.06 (0.89, 1.27)	1.05 (0.87, 1.27)	1.29 (1.07, 1.56)	.01
Women, cases (n=962)	260	226	182	171	123	
Women, HR (95% CI)	1.00	1.05 (0.88, 1.27)	1.04 (0.86, 1.26)	1.20 (0.98, 1.46)	1.21 (0.96, 1.51)	.04
**Digestive Tract**						
Men, cases (n=883)	208	180	173	167	155	
Men, HR (95% CI)	1.00	0.94 (0.77, 1.15)	1.01 (0.82, 1.24)	1.12 (0.91, 1.39)	1.32 (1.06, 1.64)	.007
Women, cases (n=751)	199	171	145	134	102	
Women, HR (95% CI)	1.00	1.03 (0.83, 1.27)	1.06 (0.85, 1.32)	1.18 (0.94, 1.48)	1.22 (0.95, 1.57)	.05
**Mouth/pharynx to small intestine**						
Men, cases (n=279)	60	60	55	57	47	
Men, HR (95% CI)	1.00	1.07 (0.74, 1.54)	1.15 (0.79, 1.68)	1.29 (0.89, 1.87)	1.26 (0.85, 1.88)	.15
Women, cases (n=174)	44	42	27	30	31	
Women, HR (95% CI)	1.00	1.27 (0.82, 1.95)	1.00 (0.61, 1.63)	1.30 (0.80, 2.11)	1.89 (1.16, 3.08)	.03
**Stomach**						
Men, cases (n=78)	11	20	13	18	16	
Men, HR (95% CI)	1.00	2.09 (0.98, 4.44)	1.58 (0.70, 3.55)	2.21 (1.02, 4.80)	2.32 (1.04, 5.15)	.05
Women, cases (n=118)	21	23	27	23	24	
Women, HR (95% CI)	1.00	1.20 (0.44, 3.30)	1.53 (0.56, 4.23)	1.92 (0.69, 5.37)	2.40 (0.81, 7.09)	.07
**Colorectum**						
Men, cases (n=606)	149	121	118	110	108	
Men, HR (95% CI)	1.00	0.88 (0.69, 1.13)	0.93 (0.72, 1.19)	1.04 (0.81, 1.35)	1.32 (1.02, 1.71)	.03
Women, cases (n=580)	156	129	118	106	71	

Women, HR (95% CI)	1.00	0.96 (0.76, 1.22)	1.06 (0.83, 1.36)	1.14 (0.88, 1.47)	1.04 (0.77, 1.40)	.43
**Digestive Accessory Organs**						
Men, cases (n=281)	66	65	65	40	45	
Men, HR (95% CI)	1.00	1.02 (0.71, 1.44)	1.19 (0.84, 1.70)	0.80 (0.53, 1.19)	1.15 (0.77, 1.70)	.85
Women, cases (n=211)	61	55	37	37	21	
Women, HR (95% CI)	1.00	1.16 (0.80, 1.68)	0.97 (0.64, 1.48)	1.29 (0.84, 1.97)	1.11 (0.66, 1.87)	.51
**Pancreas**						
Men, cases (n=220)	53	54	50	29	34	
Men, HR (95% CI)	1.00	1.03 (0.70, 1.51)	1.12 (0.75, 1.67)	0.70 (0.44, 1.11)	1.04 (0.66, 1.63)	.65
Women, cases (n=175)	51	44	31	33	16	
Women, HR (95% CI)	1.00	1.17 (0.77, 1.76)	0.99 (0.63, 1.57)	1.41 (0.90, 2.23)	1.10 (0.61, 1.99)	.41
**Liver and Gallbladder**						
Men, cases (n=61)	13	11	15	11	11	
Men, HR (95% CI)	1.00	0.96 (0.42, 2.19)	1.57 (0.73, 3.37)	1.24 (0.54, 2.85)	1.65 (0.71, 3.80)	.20
Women, cases (n=58)	18	13	9	11	7	
Women, HR (95% CI)	1.00	0.90 (0.43, 1.89)	0.80 (0.35, 1.83)	1.13 (0.51, 2.51)	1.01 (0.40, 2.55)	.92

NSAIDs=non-steroidal anti-inflammatory drugs; EDIH=empirical dietary index for hyperinsulinemia.

[*] EDIH scores were adjusted for total energy intake using the residual method. Lower scores indicate insulin sensitive diets, and higher scores indicate hyperinsulinemic diets.

[†] Heterogeneity for risk was tested using duplication method cause-specific Cox regression analyses

[‡] All analyses were adjusted for the following potential confounding variables: race, family history of cancer, history of endoscopy, multivitamin use, total alcohol intake, physical activity, pack-years of smoking, regular aspirin use, regular NSAIDs use, BMI (continuous), and additionally for menopausal status, and postmenopausal hormone use in women.

[§] The p-value for linear trend across EDIH quintiles was the p-value of the ordinal variable constructed by assigning quintile medians to all participants in the quintile. Models for linear trend were adjusted for all covariates listed in footnote #3.

Supplementary Table 4. Multivariable-adjusted hazard ratios and 95% confidence intervals for digestive system cancer risk in quintiles of the empirical dietary index for hyperinsulinemia (EDIH) scores among overweight or obese (BMI ≥ 25 kg/m^2) men and women[*,†,‡]

	Quintile 1 (reference)	Quintile 2	Quintile 3	Quintile 4	Quintile 5	P-trend[§]
**Total Digestive System**						
Men, cases (n=1005)	166	189	229	205	216	
Men, HR (95% CI)	1.00	1.01 (0.81, 1.25)	1.23 (1.00, 1.51)	1.07 (0.87, 1.33)	1.22 (0.99, 1.51)	.05
Women, cases (n=1483)	237	287	323	307	329	
Women, HR (95% CI)	1.00	1.05 (0.88, 1.25)	1.10 (0.93, 1.31)	1.07 (0.90, 1.28)	1.25 (1.05, 1.50)	.01
**Digestive Tract**						
Men, cases (n=833)	134	154	185	180	180	
Men, HR (95% CI)	1.00	1.02 (0.81, 1.30)	1.23 (0.98, 1.55)	1.17 (0.93, 1.47)	1.27 (1.00, 1.60)	.02
Women, cases (n=1108)	177	209	236	229	257	
Women, HR (95% CI)	1.00	1.02 (0.83, 1.25)	1.08 (0.88, 1.32)	1.08 (0.88, 1.32)	1.28 (1.04, 1.56)	.01
**Mouth/pharynx to small intestine**						
Men, cases (n=205)	34	41	36	43	51	
Men, HR (95% CI)	1.00	1.11 (0.70, 1.77)	0.98 (0.60, 1.58)	1.12 (0.70, 1.80)	1.28 (0.81, 2.02)	.28
Women, cases (n=253)	36	48	60	56	53	
Women, HR (95% CI)	1.00	1.11 (0.71, 1.73)	1.38 (0.90, 2.12)	1.34 (0.87, 2.08)	1.31 (0.84, 2.05)	.18
**Stomach**						
Men, cases (n=47)	6	12	8	12	9	
Men, HR (95% CI)	1.00	1.69 (0.62, 4.60)	1.20 (0.40, 3.59)	1.85 (0.66, 5.16)	1.55 (0.52, 4.58)	.46
Women, cases (n=118)	21	23	27	23	24	
Women, HR (95% CI)	1.00	0.93 (0.43, 1.99)	1.12 (0.54, 2.32)	0.92 (0.43, 1.99)	1.03 (0.48, 2.21)	.94
**Colorectum**						
Men, cases (n=630)	100	113	149	138	130	
Men, HR (95% CI)	1.00	1.01 (0.76, 1.33)	1.33 (1.02, 1.73)	1.19 (0.91, 1.56)	1.28 (0.97, 1.67)	.04
Women, cases (n=859)	142	162	176	174	205	

Women, HR (95% CI)	1.00	1.00 (0.79, 1.26)	1.00 (0.79, 1.25)	1.01 (0.80, 1.27)	1.26 (1.01, 1.59)	.04
**Digestive Accessory Organs**						
Men, cases (n=172)	32	35	44	25	36	
Men, HR (95% CI)	1.00	0.94 (0.58, 1.54)	1.22 (0.76, 1.95)	0.67 (0.39, 1.15)	1.04 (0.63, 1.72)	.79
Women, cases (n=375)	60	78	87	78	72	
Women, HR (95% CI)	1.00	1.11 (0.79, 1.56)	1.16 (0.83, 1.62)	1.06 (0.75, 1.51)	1.18 (0.82, 1.69)	.48
**Pancreas**						
Men, cases (n=346)	79	78	81	48	60	
Men, HR (95% CI)	1.00	0.75 (0.43, 1.32)	1.02 (0.59, 1.75)	0.59 (0.32, 1.08)	0.88 (0.50, 1.57)	.54
Women, cases (n=319)	52	75	63	66	63	
Women, HR (95% CI)	1.00	1.23 (0.86, 1.76)	0.95 (0.65, 1.38)	1.02 (0.70, 1.48)	1.11 (0.75, 1.64)	.95
**Liver and Gallbladder**						
Men, cases (n=46)	6	10	14	6	10	
Men, HR (95% CI)	1.00	1.83 (0.66, 5.07)	2.15 (0.79, 5.85)	0.99 (0.31, 3.17)	1.80 (0.62, 5.22)	.59
Women, cases (n=111)	19	18	27	25	22	
Women, HR (95% CI)	1.00	0.83 (0.43, 1.60)	1.18 (0.64, 2.15)	1.10 (0.59, 2.05)	1.16 (0.60, 2.22)	.47

NSAIDs=non-steroidal anti-inflammatory drugs; EDIH=empirical dietary index for hyperinsulinemia.

*EDIH scores were adjusted for total energy intake using the residual method. Lower scores indicate insulin sensitive diets, and higher scores indicate hyperinsulinemic diets.

†Heterogeneity for risk was tested using duplication method cause-specific Cox regression analyses

‡All analyses were adjusted for the following potential confounding variables: race, family history of cancer, history of endoscopy, multivitamin use, total alcohol intake, physical activity, pack-years of smoking, regular aspirin use, regular NSAIDs use, BMI (continuous), and additionally for menopausal status, and postmenopausal hormone use in women.

§The p-value for linear trend across EDIH quintiles was the p-value of the ordinal variable constructed by assigning quintile medians to all participants in the quintile. Models for linear trend were adjusted for all covariates listed in footnote #3.

Supplementary Table 5. Multivariable-adjusted hazard ratios and 95% confidence intervals for digestive system cancer risk in quintiles of the empirical dietary index for hyperinsulinemia (EDIH) scores among men and women excluding diabetics[*,†,‡]

	Quintile 1 (reference)	Quintile 2	Quintile 3	Quintile 4	Quintile 5	P-trend[§]
**Total Digestive System**						
Men, cases (n=1788)	372	354	394	329	339	
Men, HR (95% CI)	1.00	0.97 (0.84, 1.13)	1.14 (0.99, 1.32)	1.02 (0.87, 1.19)	1.24 (1.06, 1.44)	.007
Women, cases (n=2004)	421	415	406	389	373	
Women, HR (95% CI)	1.00	1.01 (0.88, 1.16)	1.05 (0.91, 1.21)	1.10 (0.96, 1.27)	1.23 (1.06, 1.42)	.003
**Digestive Tract**						
Men, cases (n=1423)	286	275	304	284	274	
Men, HR (95% CI)	1.00	1.00 (0.84, 1.18)	1.16 (0.98, 1.36)	1.15 (0.98, 1.37)	1.32 (1.11, 1.57)	<.001
Women, cases (n=1553)	319	310	314	311	299	
Women, HR (95% CI)	1.00	1.00 (0.86, 1.18)	1.07 (0.91, 1.26)	1.16 (0.99, 1.37)	1.27 (1.07, 1.50)	.001
**Mouth/pharynx to small intestine**						
Men, cases (n=360)	69	68	70	77	76	
Men, HR (95% CI)	1.00	1.00 (0.74, 1.36)	1.04 (0.76, 1.42)	1.21 (0.89, 1.64)	1.18 (0.86, 1.62)	.17
Women, cases (n=427)	80	90	87	86	84	
Women, HR (95% CI)	1.00	1.06 (0.75, 1.49)	1.18 (0.84, 1.66)	1.41 (1.01, 1.97)	1.57 (1.12, 2.22)	.003
**Stomach**						
Men, cases (n=108)	15	25	19	29	20	
Men, HR (95% CI)	1.00	1.69 (0.88, 3.24)	1.38 (0.69, 2.75)	2.14 (1.13, 4.05)	1.75 (0.88, 3.49)	.08
Women, cases (n=99)	16	17	22	23	21	
Women, HR (95% CI)	1.00	1.03 (0.52, 2.07)	1.48 (0.76, 2.86)	1.68 (0.87, 3.27)	1.82 (0.92, 3.61)	.03
**Colorectum**						
Men, cases (n=1197)	250	243	244	236	224	
Men, HR (95% CI)	1.00	0.99 (0.81, 1.21)	1.20 (0.98, 1.46)	1.11 (0.91, 1.36)	1.37 (1.12, 1.68)	.001
Women, cases (n=1439)	298	291	294	280	276	

Women, HR (95% CI)	1.00	0.99 (0.83, 1.19)	1.05 (0.88, 1.26)	1.11 (0.92, 1.33)	1.20 (0.99, 1.44)	.04
**Digestive Accessory Organs**						
Men, cases (n=451)	102	105	92	78	74	
Men, HR (95% CI)	1.00	0.90 (0.66, 1.23)	1.11 (0.82, 1.50)	0.58 (0.40, 0.83)	0.97 (0.70, 1.36)	.31
Women, cases (n=586)	121	133	124	115	93	
Women, HR (95% CI)	1.00	1.03 (0.78, 1.36)	0.98 (0.73, 1.30)	0.93 (0.68, 1.26)	1.10 (0.81, 1.51)	.80
**Pancreas**						
Men, cases (n=287)	70	62	72	35	48	
Men, HR (95% CI)	1.00	0.84 (0.59, 1.19)	1.04 (0.75, 1.46)	0.53 (0.35, 0.80)	0.83 (0.57, 1.21)	.09
Women, cases (n=381)	87	93	69	76	56	
Women, HR (95% CI)	1.00	1.14 (0.84, 1.53)	0.91 (0.66, 1.25)	1.12 (0.82, 1.54)	1.02 (0.72, 1.45)	.93
**Liver and Gallbladder**						
Men, cases (n=78)	16	17	18	10	17	
Men, HR (95% CI)	1.00	1.23 (0.61, 2.49)	1.39 (0.70, 2.79)	0.81 (0.36, 1.82)	1.76 (0.86, 3.59)	.29
Women, cases (n=126)	31	23	27	17	28	
Women, HR (95% CI)	1.00	0.70 (0.41, 1.22)	0.88 (0.52, 1.49)	0.61 (0.33, 1.11)	1.21 (0.71, 2.08)	.65

NSAIDs=non-steroidal anti-inflammatory drugs; EDIH=empirical dietary index for hyperinsulinemia.

[*] EDIH scores were adjusted for total energy intake using the residual method. Lower scores indicate insulin sensitive diets, and higher scores indicate hyperinsulinemic diets.

[†] Heterogeneity for risk was tested using duplication method cause-specific Cox regression analyses

[‡] All analyses were adjusted for the following potential confounding variables: race, family history of cancer, history of endoscopy, multivitamin use, total alcohol intake, physical activity, pack-years of smoking, regular aspirin use, regular NSAIDs use, and additionally for menopausal status, and postmenopausal hormone use in women.

[§] The p-value for linear trend across EDIH quintiles was the p-value of the ordinal variable constructed by assigning quintile medians to all participants in the quintile. Models for linear trend were adjusted for all covariates listed in footnote #3.

Supplementary Table 6. Multivariable-adjusted hazard ratios and 95% confidence intervals for digestive system cancer risk in quintiles of the empirical dietary index for hyperinsulinemia (ELIH) scores among men and women excluding diabetics[*,†, ‡]

	Quintile 1 (reference)	Quintile 2	Quintile 3	Quintile 4	Quintile 5	P-trend[§]
**Total Digestive System**						
Men, cases (n=1788)	301	307	334	371	475	
Men, HR (95% CI)	1.00	1.04 (0.88, 1.22)	1.12 (0.96, 1.31)	1.23 (1.05, 1.43)	1.52 (1.31, 1.76)	<.001
Women, cases (n=2004)	336	364	388	431	485	
Women, HR (95% CI)	1.00	1.11 (0.95, 1.29)	1.13 (0.98, 1.31)	1.22 (1.05, 1.40)	1.41 (1.23, 1.63)	<.001
**Digestive Tract**						
Men, cases (n=1432)	232	238	274	302	377	
Men, HR (95% CI)	1.00	1.05 (0.87, 1.26)	1.19 (0.99, 1.42)	1.30 (1.09, 1.55)	1.59 (1.34, 1.88)	<.001
Women, cases (n=1553)	376	267	270	311	335	
Women, HR (95% CI)	1.00	1.04 (0.88, 1.23)	1.14 (0.97, 1.35)	1.20 (1.02, 1.41)	1.36 (1.16, 1.60)	<.001
**Mouth/pharynx to small intestine**						
Men, cases (n=418)	66	72	97	76	107	
Men, HR (95% CI)	1.00	1.11 (0.79, 1.56)	1.47 (1.07, 2.02)	1.15 (0.83, 1.61)	1.49 (1.09, 2.03)	.02
Women, cases (n=360)	66	58	64	82	90	
Women, HR (95% CI)	1.00	0.93 (0.65, 1.33)	0.99 (0.70, 1.40)	1.24 (0.89, 1.72)	1.43 (1.03, 1.97)	.006
**Stomach**						
Men, cases (n=108)	19	20	25	21	23	
Men, HR (95% CI)	1.00	1.12 (0.59, 2.11)	1.35 (0.74, 2.48)	1.24 (0.66, 2.34)	1.23 (0.66, 2.29)	.49
Women, cases (n=99)	17	12	17	24	29	
Women, HR (95% CI)	1.00	0.68 (0.32, 1.43)	0.92 (0.47, 1.81)	1.27 (0.68, 2.39)	1.50 (0.82, 2.77)	.04
**Colorectum**						
Men, cases (n=1008)	167	167	177	226	271	
Men, HR (95% CI)	1.00	1.03 (0.83, 1.28)	1.06 (0.86, 1.32)	1.36 (1.11, 1.66)	1.62 (1.33, 1.98)	<.001
Women, cases (n=1197)	202	212	247	255	281	

Women, HR (95% CI)	1.00	1.07 (0.88, 1.30)	1.19 (0.99, 1.43)	1.19 (0.99, 1.44)	1.35 (1.12, 1.62)	<.001
**Digestive Accessory Organs**						
Men, cases (n=365)	69	69	60	69	98	
Men, HR (95% CI)	1.00	0.99 (0.70, 1.39)	0.91 (0.64, 1.29)	1.00 (0.71, 1.40)	1.30 (0.95, 1.78)	.08
Women, cases (n=451)	69	94	77	96	115	
Women, HR (95% CI)	1.00	1.36 (0.99, 1.86)	1.09 (0.79, 1.51)	1.29 (0.95, 1.77)	1.60 (1.18, 2.17)	.005
**Pancreas**						
Men, cases (n=346)	79	78	81	48	60	
Men, HR (95% CI)	1.00	1.03 (0.71, 1.50)	0.98 (0.67, 1.44)	0.92 (0.63, 1.35)	1.14 (0.79, 1.63)	.61
Women, cases (n=494)	103	119	94	99	79	
Women, HR (95% CI)	1.00	1.32 (0.95, 1.83)	1.05 (0.74, 1.48)	1.10 (0.78, 1.54)	1.50 (1.09, 2.07)	.04
**Liver and Gallbladder**						
Men, cases (n=78)	14	11	7	17	29	
Men, HR (95% CI)	1.00	0.81 (0.36, 1.80)	0.57 (0.23, 1.43)	1.35 (0.66, 2.78)	2.03 (1.05, 3.91)	.005
Women, cases (n=126)	18	21	21	31	35	
Women, HR (95% CI)	1.00	1.14 (0.61, 2.15)	1.10 (0.58, 2.07)	1.55 (0.86, 2.79)	1.81 (1.02, 3.22)	.02

NSAIDs=non-steroidal anti-inflammatory drugs; ELIH=empirical lifestyle index for hyperinsulinemia.

*ELIH scores were adjusted for total energy intake using the residual method. Lower scores indicate insulin sensitive lifestyles, and higher scores indicate hyperinsulinemic lifestyles.

†Heterogeneity for risk was tested using duplication method cause-specific Cox regression analyses

‡All analyses were adjusted for the following potential confounding variables: race, family history of cancer, history of endoscopy, multivitamin use, total alcohol intake, pack-years of smoking, regular aspirin use, regular NSAIDs use, and additionally for menopausal status, and postmenopausal hormone use in women.

§The p-value for linear trend across ELIH quintiles was the p-value of the ordinal variable constructed by assigning quintile medians to all participants in the quintile. Models for linear trend were adjusted for all covariates listed in footnote #3.
